# Comparative analysis of the phase III clinical trials of anti-PD1 monotherapy in head and neck squamous cell carcinoma patients (CheckMate 141 and KEYNOTE 040)

**DOI:** 10.1186/s40425-019-0578-0

**Published:** 2019-04-03

**Authors:** Sara I. Pai, Sandrine Faivre, Lisa Licitra, Jean-Pascal Machiels, Jan B. Vermorken, Paolo Bruzzi, Viktor Gruenwald, Raul E. Giglio, C. René Leemans, Tanguy Y. Seiwert, Denis Soulieres

**Affiliations:** 1 Division of Surgical Oncology, Department of Surgery, Massachusetts General Hospital Cancer Center, Harvard Medical School, Boston, MA USA; 2 Oncologie Médicale, Hôpitaux Universitaires Paris Nord Val de Seine, Clichy, France; 3 Fondazione IRCCS Istituto Nazionale Tumori Milano, Università degli Studi Milano, Milan, Italy; 4 Service d’Oncologie Médicale, Institut Roi Albert II, Cliniques Universitaires Saint-Luc and Institut de Recherche Clinique et Expérimentale, UCLouvain, Brussels, Belgium; 5 Department of Medical Oncology, Antwerp University Hospital, Edegem, Belgium; 6 Unit of Clinical Epidemiology, National Cancer Research Institute, San Martino, IST Hospital, Genoa, Italy; 7 Clinic for Internal Medicine (Tumor Research) and Clinic for Urology, University Hospital Essen, Essen, Germany; 8 Funcional de Tumores de Cabeza y Cuello, Instituto de Oncología Ángel H. Roffo, Universidad de Buenos Aires, Buenos Aires, Argentina; 9 Department of Otolaryngology, Head and Neck Surgery, Amsterdam University Medical Centers, Amsterdam, The Netherlands; 10 Department of Medicine, Section of Hematology/Oncology, University of Chicago, Chicago, IL USA; 11 Département Hématologie-oncologie, Centre hospitalier de l’Université de Montréal, Montréal, Canada

**Keywords:** Head and neck squamous cell carcinoma, Anti-PD-1 therapy, Immune checkpoint therapy, Immunotherapy, Clinical trials

## Abstract

Two phase III clinical trials (CheckMate 141 and KEYNOTE 040) have independently demonstrated that overall survival (OS) in recurrent and/or metastatic head and neck squamous cell carcinoma (R/M HNSCC) patients, who have failed platinum-based therapy, can be improved with anti-PD1 monotherapy. Treatment with nivolumab or pembrolizumab in R/M HNSCC patients led to an improved OS with a hazards ratio (HR) of 0.70 (95%CI 0.51–0.96; *p* = 0.01) and HR of 0.80 (95%CI 0.65–0.98, *p* = 0.0161), respectively, as compared to standard of care (SOC) chemo monotherapy regimens (specifically, cetuximab, docetaxel, or methotrexate). The gain in OS was similar in both studies, underscoring the role of anti-PD1 drugs in R/M HNSCC patients. One of the striking discrepancies between CheckMate 141 and KEYNOTE 040 was the OS observed in the control SOC arms (6.9 months median in KEYNOTE 040 versus 5.1 months in CheckMate 141), which inadvertently set a higher threshold in the bio-statistical analysis of KEYNOTE 040 so that the clinical outcome of every patient was influential in the analysis. We perform a comparative analysis of the two studies to identify potential factors in the control arm that can impact clinical trial bio-statistical outcomes and which may have implications for future immunotherapy clinical trial designs.

## Overview of the phase III clinical trials

In the CheckMate 141 clinical trial sponsored by Bristol-Myers Squibb (BMS) (NCT02105636), 361 recurrent and/or metastatic (R/M) head and neck squamous cell carcinoma (HNSCC) patients were randomized 2:1 to either nivolumab (*N* = 240) or standard of care (SOC) chemo monotherapy (*N* = 121) [1]. The monotherapy treatment options included weekly applications of either methotrexate 40 mg/m^2^, docetaxel 30 mg/m^2^, or cetuximab 250 mg/m^2^ (400 mg/m^2^ loading dose first). The patients in the study were stratified based on prior cetuximab treatment only. In the KEYNOTE 040 clinical trial sponsored by Merck Sharp and Dohme (MSD) (NCT02252042), 495 R/M HNSCC patients were randomized 1:1 to either pembrolizumab (*N* = 247) or SOC chemo monotherapy (*N* = 248) [2–4]. The monotherapy treatment options included methotrexate 40 mg/m^2^ weekly, docetaxel 75 mg/m^2^ every 3 weeks, or cetuximab 250 mg/m^2^ weekly (400 mg/m^2^ loading dose first). Stratification factors included p16 status and a tumor proportion score (TPS) of PDL1 expression ≥ 50% or < 50%. In both of these studies, the primary bio-statistical endpoint was overall survival (OS) in the intention-to-treat (ITT) population.

## Factors impacting overall survival in the control arms

One of the main differences between these two clinical trials was the OS observed in the control SOC arms (6.9 months median in KEYNOTE 040 versus 5.1 months in CheckMate 141). The 1.8-month difference in median OS in the control arms may have had significant bio-statistical implications for the endpoint analysis.

### Patient selection

There were two important differences in the selection of patients between these two trials. Although both trials included patients with platinum refractory R/M HNSCC, KEYNOTE 040 excluded patients who recurred or progressed within 3 months of previous multimodal therapy containing platinum for locally advanced disease. Thus, KEYNOTE 040 was excluding the rapidly progressing patient population from the trial. In addition, in KEYNOTE 040, only 1.2% (*N* = 6 of 495) of the HNSCC patients had received ≥ 3 prior lines of therapy as compared to 19.9% (*N* = 72 of 361) in CheckMate141. Thus, in KEYNOTE 040, the population was less heavily pre-treated and aggressive tumor growth characteristics were excluded from the trial and these exclusion criteria most likely contributed to the improved OS observed in the control arm. This inadvertently set a higher threshold in the bio-statistical analysis of KEYNOTE 040 so that the clinical outcome of every patient was influential in the analysis.

### Differential distribution of SOC treatment regimens

In addition, even though the mono chemotherapy treatment options in the SOC arms were the same in both clinical trials, there were differences in the dosing and the overall distribution of the patients in the SOC treatment regimen received. Specifically, there were differences in the dosing of docetaxel. In the KEYNOTE 040 trial, docetaxel was given at 75 mg/m^2^ q3weeks whereas in CheckMate 141 the docetaxel dose was 30 mg/m^2^ qweek. Whether this difference in dosing of docetaxel makes a difference is unclear but we may speculate that the q3week dosing may be reserved for more robust patients in KEYNOTE040. In addition, docetaxel q3 weeks has been reported as slightly more efficient in terms of response rate or survival than the weekly schedule in other sensitive tumor types, such as breast and prostate cancer [5, 6]. There was also a higher percentage of patients who were treated with docetaxel (42% versus 21%) and cetuximab (30% versus 11%) in KEYNOTE 040 as compared to CheckMate 141, respectively. In contrast, in CheckMate 141, there was a higher number of patients who received methotrexate (38% versus 27% in KEYNOTE 040). While the CheckMate 141 trial was not designed to compare the three regimens used in the SOC arm, docetaxel appeared to result in a slightly improved OS as compared to patients receiving methotrexate and cetuximab, although the overall numbers were small to be able to make definitive conclusions. Thus, the difference in the number of R/M HNSCC patients receiving docetaxel in KEYNOTE 040 as compared to methotrexate in CheckMate 141 may have also contributed to the improved OS in the control arms (6.9 months versus 5.1 months).

### Subsequent treatment with immune checkpoint inhibitors in the SOC arm

Both phase III trials had a subset of patients who were allocated to the SOC arm but did not receive SOC therapy. In KEYNOTE 040, 5.6% (*N* = 14 of 248) of patients as compared to 8.2% (*N* = 10 of 121) of patients in CheckMate 141 did not receive SOC therapy. Most importantly, the treatment option provided at the time of progression was very different between the two clinical trials based on the timing of the studies and most likely had a large influence in the OS observed in the control arms. At the time of the KEYNOTE 040 study, **both** pembrolizumab and nivolumab were Food and Drug Administration (FDA) approved for the treatment of R/M HNSCC patients in contrast to when the CheckMate 141 study had completed, in which an immune checkpoint inhibitor (ICI) was only accessible through clinical trials. In CheckMate 141, the data cutoff point for the analyses of OS, progression free survival (PFS), and safety was December 18, 2015 with a median duration of follow-up for OS of 5.1 months (range, 0 to 16.8) and data on rate of response was based on a cutoff of May 5, 2016. Pembrolizumab had received FDA approval in August of 2016 and nivolumab in November of 2016. KEYNOTE 040 had a data cutoff point of May 15, 2017. Correspondingly, in KEYNOTE 040, 12.5% (31 of 248) of patients in the SOC arm received an ICI at the time of clinical progression. The impact of receiving subsequent ICI in the SOC arm had a significant impact on median OS, with patients in the SOC arm receiving subsequent ICI having a prolonged median OS of 20.1 months (*N* = 15, 95% CI: 14.0-NE months) as compared to a median OS of 9.7 months (*N* = 58, 95% CI: 9.0–11.3 months) in patients who went on to receive a non-ICI therapy or a median OS of 4.5 months (*N* = 134, 95% CI: 3.7–5.0 months) in patients who received no subsequent therapy.

### Patient selection based on PD-L1 expression

These trials also highlight the importance of patient selection in clinical trial design and raises the question whether patient selection based on expression of a prognostic biomarker, such as PD-L1, should be considered as an eligibility criteria for future PD-1/PD-L1 targeted clinical trials. Both of the phase III clinical studies provide insight into this question through a sub-analysis performed on OS based on PD-L1 expression > 1% within the tumor microenvironment. In KEYNOTE 040, using a PD-L1 combined positive score (CPS) of ≥ 1, which is defined by PD-L1 expression on both immune and tumor cells, the HR was 0.74 (95% CI: 0.58–0.93, *p* = 0.0049) with a median OS of 8.7 months with anti-PD1 treatment. Similarly, in CheckMate 141, a PD-L1 tumor cell expression score of ≥ 1% resulted in a HR of 0.55 (95% CI: 0.36–0.83) with a median OS of 8.7 months. If PD-L1 expression serves as a true prognostic biomarker of response for PD-1 therapy, an increasing percentage of PD-L1 expression within the tumor microenvironment should correlate with improved OS and this is indeed what is observed. In KEYNOTE 040, a PD-L1 tumor proportion score (TPS) of ≥ 50% improved the HR to 0.53 (95% CI: 0.35–0.81, *p* = 0.0014) with a median OS of 11.6. Correspondingly, the complete response (CR) rates also increase with PD-L1 expression. In KEYNOTE 040, the overall CR rate in the pembrolizumab treated group was 1.6% (*N* = 4 of 247). However, in patients with a CPS ≥ 1 the CR was 2.0% (*N* = 4 of 196), and in patients with a TPS ≥ 50%, CR was 4.7% (*N* = 3 of 64). Furthermore, PFS and RR also increased with increasing PD-L1 expression (Table 1).

**Table 1:** Comparison of key subgroups in Checkmate 141 and KEYNOTE 040 trials

	Nivolumab	SOC	SOC	Pembrolizumab
	*n* = 240	*n* = 121	*n* = 248	*n* = 247
≥3 prior lines	19,9		1,2	
PD-L1 TPS ≥ 1% (%)	37,0	50,0	77,0	79,4
PD-L1 TPS ≥ 50% (%)	–	–	26,2	25,9
HPV+ (%)	26,0	24,0	23,4	24,7
ECOG-0 (%)	20,0	19,0	17,0	19,0
ORR (%)	13,0	6,0	10,1	14,6
mPFS (mo.)	2,0	2,3	2,3	2,1
OS HR (CI95%)	0,70 (0,51-0,96)		0,80 (0,65-0,98)	
	P = 0,01		P = 0,0161	
mOS (mo.)	7,5	5,1	6,9	8,4
1y-OS rate	0,4	0,2	0,3	0,4

## Conclusion

In the initial reported protocol-specified final analysis, the KEYNOTE 040 study did not meet their bio-statistical primary endpoint of OS [2]. However, the survival data of a pending 12 patients, with no change to the duration of the follow-up, made the difference in the positive outcome reported in the protocol-specified final analysis of KEYNOTE 040 [3, 4]. Although inter-study comparisons must be put in the context of hidden biases which always exist in trials, confounding factors which may influence the clinical outcomes of the control arm will continue to be a challenge as we move toward combinatorial immunotherapy trials and calls upon innovative bio-statistical approaches and clinical trial design. The observed clinical benefit with anti-PD1 therapies in patients expressing PD-L1 within the tumor microenvironment, may call for paradigm shifts to include biomarker expression as part of primary or secondary bio-statistical endpoints in future clinical trials, rather than as exploratory endpoints as traditionally done. This is particularly important, since an increasing number of CRs is being observed in the biomarker expressing patient populations. Furthermore, given the impact on costs and tolerability, patient selection has to take place in order to provide individualized treatment, sparing toxicities and maximizing clinical outcome. Immunotherapy when applied in the appropriate setting has the potential to change the lives of cancer patients and affords us further opportunities to explore personalized treatment plans for our patients.

## Abbreviations

BMS: Bristol-Myers Squibb
CPS: Combined positive score
CR: Complete response
FDA: Food and Drug Administration
HNSCC: Head and neck squamous cell carcinoma
HR: Hazards ratio
ICI: Immune checkpoint inhibitors
ITT: Intention-to-treat
MSD: Merck Sharp and Dohme
OS: Overall survival
R/M: Recurrent and/or metastatic
SOC: Standard of care
TPS: Tumor proportion score
